# The Aphasia Physical EXercise (APEX) study: feasibility of a new high-intensity functional interval training for stroke survivors with aphasia

**DOI:** 10.1080/02687038.2026.2703146

**Published:** 2026-08-04

**Authors:** Maria V. Ivanova, Michelle Gravier, Christian Thompson, Erica Pitsch, Cathra Halabi, Nina F. Dronkers, Vanessa Anderson, Ferdaus Ghafury

**Affiliations:** a Department of Psychology, University of California Berkeley, Berkeley, CA, USA;; b Department of Speech, Language and Hearing Sciences, California State University East Bay, Hayward, CA, USA;; c Department of Kinesiology, University of San Francisco, San Francisco, CA, USA;; d Department of Physical Therapy and Rehabilitation Science, University of California San Francisco, San Francisco, CA, USA;; e Department of Neurology, University of California San Francisco, San Francisco, CA, USA;; f Weill Institute for Neurosciences, University of California San Francisco, San Francisco, CA, USA

**Keywords:** Post-stroke aphasia, physical exercise, high-intensity interval training, stroke rehabilitation, language recovery

## Abstract

**Background:**

Converging evidence suggests that physical exercise promotes motor and cognitive recovery after stroke, yet its potential to support language outcomes in people with aphasia remains largely unexplored. Additionally, there is a lack of structured, high-intensity and accessible exercise programs that foster adherence among stroke survivors.

**Aims:**

This pilot study evaluated the feasibility and explored the preliminary effects of a novel High-Intensity Functional Interval InterVention (HI-FIIV) program for stroke survivors with aphasia.

**Methods & Procedures:**

This Phase 1, proof of concept, pilot feasibility study employed a single-group, pre-test/post-test observational quantitative design. Seven individuals with mild-to-moderate aphasia completed an 8-week, thrice-weekly HI-FIIV intervention. Safety, adherence, and satisfaction were documented. Pre- and post-intervention assessments included standardized measures of language, cognition, and physical fitness.

**Outcomes & Results:**

The program was safe, well tolerated, and achieved high adherence (97.9% attendance). Participants reported high satisfaction (mean 9.6/10). Positive changes were observed on several higher-level language measures, including sentence comprehension and fluency, as well as on cognitive tasks targeting selective attention and working memory. Aerobic endurance increased significantly, and greater fitness gains were associated with larger changes in fluency and comprehension.

**Conclusions:**

This feasibility trial demonstrated that people with aphasia can safely participate in high-intensity group exercise and show patterns of selective improvement in language and cognition. While preliminary, the observed relationship between cardiovascular endurance and specific improvements in language suggests that exercise-induced gains in aerobic fitness may support recovery in post-stroke aphasia. Overall, by targeting physical, cognitive, and linguistic domains simultaneously, the HI-FIIV program represents a promising candidate for further investigation as an augmentative approach for post-stroke rehabilitation.

## Introduction

There is mounting evidence that physical exercise can promote recovery post-stroke, with studies revealing a positive impact of physical exercise interventions on general health, motor and cognitive outcomes in stroke survivors (Cumming et al., 2012; Mayer et al., 2021; Ploughman & Kelly, 2016; Saunders et al., 2020; Vanderbeken & Kerckhofs, 2017). Recent clinical recommendations emphasize the need to incorporate physical fitness training into routine stroke care (Billinger et al., 2014; Mackay-Lyons et al., 2020; Saunders et al., 2014). Despite strong evidence that physical exercise has the potential to directly impact language and cognition, only one small study (Harnish et al., 2018) has thus far reported a physical exercise intervention to specifically augment recovery in people with post-stroke aphasia. The current study aims to address this gap by (1) proposing a new exercise program designed specifically for stroke survivors and (2) presenting results from a pilot study using this novel program to promote physical fitness, cognitive and language gains in post-stroke aphasia recovery.

To date many studies looked at the effect of exercise on cognitive outcomes in stroke recovery (for reviews see Cumming et al., 2012; Saunders et al., 2020; Vanderbeken & Kerckhofs, 2017), but very few have examined its impact specifically on language outcomes. General exercise intervention studies with stroke survivors have not used detailed performance-based language tests, with assessments being limited to screening tests or self-report questionnaires (for review see Mayer et al., 2021). Of the studies that measured language in stroke participants, two reported a significant increase in language abilities following exercise using the self-report scores from the communication domain of the Stroke Impact Scale (Ezeugwu & Manns, 2018; Macko et al., 2008). In another stroke study, activating physiotherapy that included physical exercise and elements of cognitive training led to significant improvements on a summary language score comprising comprehension, naming, and verbal fluency measures (Pyäriä et al., 2007). However, it is unclear how many patients with aphasia participated in those studies. Overall, across all the stroke studies looking at the impact of physical exercise on cognitive outcomes, only two specified how many participants had aphasia (Pallesen et al., 2019; Ploughman et al., 2019), with a total of only 21 participants with aphasia included. Finally, only one published study to date investigated the impact of exercise on language outcomes specifically in post-stroke aphasia and focused exclusively on naming ability. In this study Harnish et al. (2018) showed that speech-language therapy (SLT) led to greater improvements in word retrieval when it was delivered together with exercise training for 2 weeks compared to an SLT-only condition. The results also demonstrated that aerobic exercise had a more pronounced effect on naming abilities compared to an intervention involving stretching; although the small groups (*n* = 5 and 2) greatly limit generalizability, they are consistent with early-stage feasibility work in this area. There remains a significant gap in the current literature regarding the effects of exercise interventions on outcomes in post-stroke aphasia. However, emerging evidence from studies in other populations and related fields suggests that exercise interventions may enhance language recovery and amplify the benefits of traditional SLT for individuals with aphasia. Despite this promising potential, the impact of exercise on behavioral outcomes in post-stroke aphasia has yet to be systematically explored.

Beyond behavioral findings, converging evidence from basic and clinical neuroscience suggests that aerobic exercise may directly modulate neurobiological mechanisms relevant to post-stroke recovery. Aerobic exercise increases cerebral blood flow and promotes angiogenesis, thereby supporting metabolic demands in perilesional and connected brain regions (Lucas et al., 2015; Smith & Ainslie, 2017). Exercise has also been shown to stimulate the release of neurotrophic factors, including brain-derived neurotrophic factor (BDNF) and insulin-like growth factor-1 (IGF-1), which play critical roles in synaptic plasticity, neurogenesis, and cortical reorganization (Erickson et al., 2011; Mang et al., 2013; Ploughman & Kelly, 2016).

Given that language recovery in chronic post-stroke aphasia depends on experience-dependent neuroplasticity within distributed frontotemporal and domain-general control networks (Geranmayeh et al., 2014; Kiran & Thompson, 2019), exercise-induced enhancements in neurovascular function and plasticity may provide a biological substrate capable of amplifying behavioral recovery. Furthermore, executive control and working memory, domains commonly showing improvements following exercise, are known contributors to language production and comprehension (Ivanova et al., 2015; Schumacher et al., 2022). Thus, physical exercise may support aphasia recovery both through direct neurobiological mechanisms and indirectly via improvements in domain-general cognitive systems that scaffold language processing.

One of the barriers to exploring the impact of exercise on aphasia recovery is the significant variability in the types of physical exercise interventions used in general stroke recovery studies. These interventions range from low-intensity stretching (Harnish et al., 2018; Tang et al., 2016) to moderate aerobic exercise (Harnish et al., 2018) to high-intensity workouts (Gjellesvik et al., 2021). Many studies rely on standard exercise equipment (Deijle et al., 2022) or individually tailored routines (Lapointe et al., 2023; Zhang et al., 2024; Zhou et al., 2024), making replication of findings and further dissemination of these programs challenging. Furthermore, directly implementing typical exercise routines from healthy control studies with stroke survivors is difficult due to common issues like deconditioning and motor impairments, which require substantial adaptations to exercise plans.

Despite this variability, recent stroke studies highlight the importance of exercise intensity in motor recovery (Henderson et al., 2022; Klassen et al., 2020). Specifically, high-intensity interval training (HIIT) – which involves short, intense bursts of cardiovascular work followed by brief rest periods – has shown promising results for eliciting behavioral changes in stroke survivors (Crozier et al., 2018; Gjellesvik et al., 2021). A comprehensive meta-analysis published in 2024 further concluded that HIIT is the most effective intervention for achieving significant gains across various stroke recovery outcomes (Moncion et al., 2024). Importantly, post-stroke studies demonstrate that higher-intensity aerobic exercise can upregulate circulating BDNF and IGF-1 levels and that such increases have been associated with behavioral improvements (Ashcroft et al., 2022; Hsu et al., 2021), pointing to a potential mechanism behind the observed changes. Overall, these findings align with clinical recommendations emphasizing that task-specific practice at higher cardiovascular intensities may be critical for optimizing post-stroke motor recovery (Donnellan-Fernandez et al., 2022; Hornby et al., 2020; Luo et al., 2020; Mackay-Lyons et al., 2020).

Similar to motor recovery, emerging data suggests that higher intensity workouts lead to more pronounced cognitive benefits (Boyne et al., 2017; Crozier et al., 2018; Gibala et al., 2012; Gjellesvik et al., 2021; Harnish et al., 2018; Ploughman et al., 2019). For example, stroke survivors who were assigned to an aerobic exercise group achieved greater cognitive gains relative to a control group receiving non-aerobic exercise training (El-Tamawy et al., 2014; Liu-Ambrose et al., 2022; Moore et al., 2015; Yeh et al., 2019). Also, the amount of improvement in physical fitness has been directly related to the amount of cognitive gains (Kluding et al., 2011; Marzolini et al., 2012), again pointing to intensity as a critical component of an effective physical exercise intervention in terms of eliciting both physical and cognitive changes. Finally, strength and balance trainings have been shown to have positive effects on cognitive outcomes in the aging population (Dorner et al., 2007). Recent reviews of the stroke literature propound that there is a positive synergistic effect of combining aerobic exercise with resistance/strength training regimens for both cardiorespiratory and strength recovery (Marzolini et al., 2018) and cognitive outcomes (Hasan et al., 2016; Mayer et al., 2021; Saunders et al., 2020). Still, there is currently no established, detailed and widely-available physical exercise intervention that simultaneously incorporates cardiorespiratory, muscle strength and balance training elements while being accessible for chronic stroke patients with various motor, cognitive and language deficits (Mayer et al., 2021; Moncion et al., 2020). In all, there is a lack of high-intensity and accessible exercise programs that foster adherence among stroke survivors.

The current study aims to address this need by piloting a new high-intensity intervention for stroke survivors with aphasia – High-Intensity Functional Interval InterVention (HI-FIIV). This intervention, based on published research and clinical practice recommendations, is a HIIT full-body circuit training program optimized to accommodate the range of motor abilities and general deconditioning observed in stroke survivors. We specifically developed this new exercise program to provide a safe, stroke- and aphasia-friendly physical exercise intervention to achieve optimal physical fitness, cognitive and language gains.

The goal of the current Phase 1, proof of concept, pilot feasibility study in stroke survivors with aphasia was three-fold: (1) to establish the safety, adherence, and satisfaction with the new HI-FIIV program; (2) to explore its potential impact on language and cognition; and (3) to examine the relationship between gains in physical fitness and improvements in language and cognitive abilities. We hypothesized that the intervention would be safe, with high adherence and satisfaction rates. Next, we expected to see positive changes in language and cognitive abilities following the intervention. Given the exploratory nature of this study, analyses of language and cognitive outcomes were intended to identify preliminary patterns of change rather than to test definitive efficacy hypotheses. Finally, we anticipated that there would be a positive relationship between gains in physical fitness and improvements in language and cognitive abilities.

## Method

### Participants

Participant inclusion criteria were: aphasia following ischemic or hemorrhagic stroke; at least 6 months from the last stroke; proficient in English before the stroke; at least 8 years of education; between the ages of 18 and 80; no prior neurological illness (other than stroke); no significant visual or hearing disabilities. To participate in the exercise program individuals with aphasia had to be independently ambulatory, with the use of a single-point cane permitted. They also had to be medically stable with no severe and uncontrolled metabolic or cardiorespiratory conditions as determined by PAR-Q questionnaire (Adams, 1999) at study intake. Inclusion criteria further required medical clearance for exercise from their healthcare provider and termination of any individual or intensive speech-language therapy for the duration of the study.

Seven individuals with aphasia (5F/2 M, *M* = 56.3 ± 7 years) were recruited to participate in an eight-week-long, three-times-a-week physical exercise intervention. All participants were right-handed and were proficient speakers of English prior to stroke. Time post-onset varied from 11 to 241 months (*M* = 75.7 ± 77 months). Participants had post-stroke aphasia due to single left (*n* = 3) or right (*n* = 1) hemisphere stroke, multiple bilateral strokes (*n* = 2), or a left hemisphere stroke combined with diffuse bilateral hypoxic injury due to cardiac arrest (*n* = 1). All participants had aphasia as indicated by an Aphasia Quotient on the Western Aphasia Battery (WAB; Kertesz, 2007; *M* = 82 ± 7). See Table 1 for individual participant characteristics.

Prior to recruitment, the study was approved by the authors’ Institutional Review Boards. All participants provided written informed consent before participation. The study was registered on ClinicalTrials.gov (identifier: NCT06185023).

### Study design

This Phase 1, proof of concept, pilot feasibility study employed a single-group, pre-test/post-test observational quantitative design. Participants completed a battery of language, cognitive, and functional fitness assessments one week prior to and one week following the 8-week exercise intervention. The intervention consisted of a novel HIIT program specifically developed by the authors for individuals with post-stroke impairments (see Section 2.3 for details). The program was delivered in person in a group format three times per week (Monday, Wednesday, and Friday) over eight weeks, totaling 24 sessions. Each session lasted 40–50 minutes and was led by a certified fitness instructor.

### Physical exercise intervention

The High-Intensity Functional Interval InterVention (HI-FIIV) used in this study is a HIIT program designed to accommodate varied motor abilities and general deconditioning commonly observed in stroke survivors. HI-FIIV is based on published research and clinical practice recommendations (Anjos et al., 2022; Mackay-Lyons et al., 2020). We have previously piloted a very similar exercise program in healthy older adults and found it to be safe and feasible, with no adverse events, no dropouts, and high compliance. This HIIT program offered a promising alternative to traditional cardiovascular exercise modalities for older adults with additional benefits for functional fitness and cognition (Anderson et al., 2024). The current study is the first to test its safety and feasibility in individuals with post-stroke aphasia.

**Figure 1.**
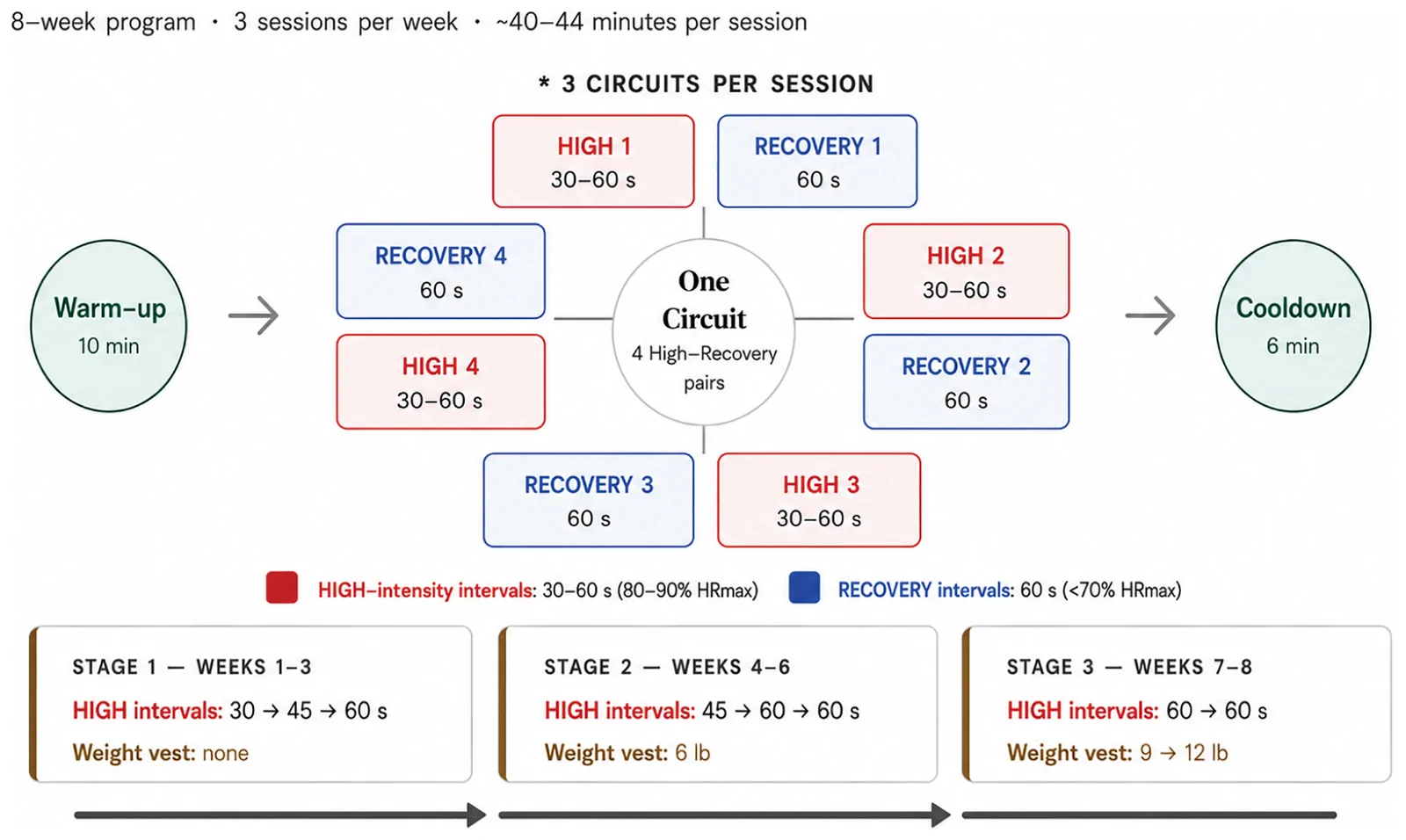
Structure of the HI-FIIV intervention. The intervention consisted of an 8-week program with three sessions per week, each comprising a 10-minute warm-up, three circuits of four high-intensity/recovery interval pairs, and a 6-minute cooldown. Individual maximum heart rate (HRmax) was determined by a maximal aerobic capacity test before the intervention and monitored continuously during exercise using a Polar H10 chest strap with real-time visual feedback. Recovery intervals were held constant at 60 s throughout the intervention, whereas high-intensity interval duration progressed during stages 1 and 2 and plateaued at 60 s during Stage 3. A weighted vest was introduced in Week 4 and progressively increased during Weeks 7–8.

Each session follows a structured format: a 10-minute warm-up period is followed by a circuit of four high-intensity, full-body exercises performed for 30 to 60 seconds (with duration increasing over the course of the intervention), interleaved with four postural stability recovery exercises, each lasting 60 seconds (with duration held constant). This sequence is repeated three times per session (with a total duration of 22 to 28 minutes) and concludes with a 6-minute cooldown period. An overview of the intervention is shown in Figure 1, while a complete list of exercises included in the intervention and corresponding durations is provided in the Appendix in Table A1.

HI-FIIV utilizes a multimodal progressive HIIT design, emphasizing cardiovascular fitness and muscle strength in high-intensity intervals, and balance and mobility in recovery intervals. The high-intensity characteristic of the training is designed to increase cardiovascular fitness, improve muscle strength, and motor performance, and, most importantly, maximize cognitive and language gains (Crozier et al., 2018; Gjellesvik et al., 2021). The active recovery intervals during each HIIT training session reduce the likelihood of venous pooling, orthostatic hypotension and falls (Rodriguez et al., 2017). Additionally, low-intensity balance and sensory training exercises during these active recovery intervals are intended to address the motor control deficits affecting some patients and may elicit adaptations to physical measures beyond those for cardiovascular fitness (Gjellesvik et al., 2021). The reduction in work-recovery intervals over the course of the intervention is intended to maintain the challenge of the training program as participants improve exercise tolerance and cardiovascular fitness. There were also modest increases in movement complexity of all exercises at Week 4 and 7 of the intervention to mirror participants’ potential improvements in motor performance (see Table A1). Additionally, at Week 4 we added weighted vests weighing 6lb to increase cardiovascular demand as participants’ fitness and exercise tolerance increased. Subsequently, we increased the weight of the vests by 3lb at Week 7 and Week 8 by adding additional weight packs, reaching the final weight of 12lb (see Figure 1). To accommodate individuals with hemiparesis we intentionally avoided using any weights or resistance bands that would require single-hand gripping. For further details about the structure of our exercise intervention see Thompson et al. (2026).

**Figure 2.**
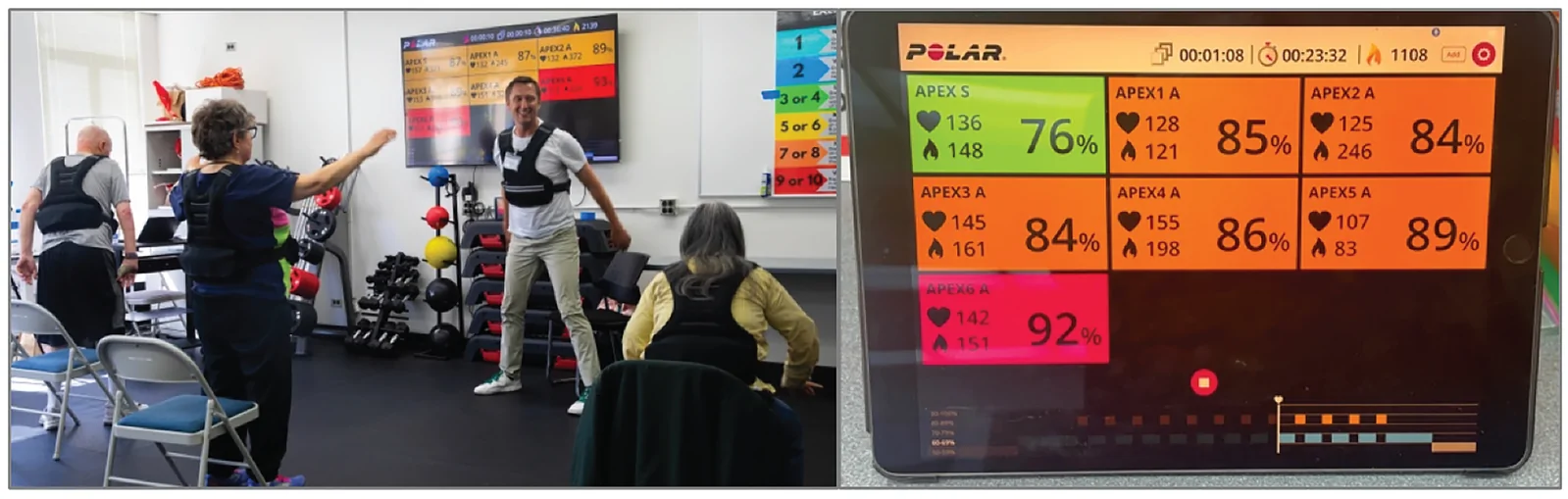
Setup of the exercise class. Participant’s HRs were projected on the big screen from an iPad, enabling everyone to clearly see their HR in real time and whether they were in the target HR zone. Note the use of weighted vests to evenly distribute the weight and accommodate participants with motor deficits.

A novel and important feature of the exercise intervention was the use of real-time heart rate (HR) monitoring and feedback. HR was measured continuously using a chest strap equipped with a Polar H10 sensor. Participants received immediate visual feedback on their HR and target zones via the Polar Coach App, which projected real-time HR data onto a large screen in the exercise room (see Figure 2 for the setup of the class and how the HR monitoring looked like). This system served two key purposes: it promoted participant safety by preventing excessive exertion and ensured that target intensities for the intervention were consistently achieved. During HI intervals, participants were instructed to maintain a HR between 80% and 90% of their individual maximum HR, as determined by a maximal aerobic capacity test conducted prior to the intervention. During recovery intervals, participants targeted HR below 70% of maximum. A detailed analysis of the HR data collected during the sessions is beyond the scope of this paper and is reported separately (Thompson et al., 2026).

### Outcome measures

**Safety** was evaluated based on the occurrence of adverse events and any exercise sessions that were prematurely terminated due to participant complaints. **Adherence** was measured by session attendance rates. **Participant satisfaction** with the intervention was assessed through an anonymous post-intervention survey, in which participants rated their enjoyment of the exercise classes on a 10-point Likert scale.

To evaluate treatment effects, participants completed a battery of outcome measures focused on language, cognition, and physical fitness both before and after the HI-FIIV program. Outcome measures were selected to capture both global language severity and specific cognitive-linguistic domains hypothesized to be responsive to high-intensity physical exercise. Prior stroke and aging studies have consistently reported exercise-related improvements in executive functions, attention, processing speed, and working memory (Kluding et al., 2011; Marzolini et al., 2012; Mayer et al., 2021; Saunders et al., 2020). Accordingly, our cognitive battery was designed to sample these core domains using well-established experimental paradigms.

Language measures were chosen to reflect both overall aphasia severity and more effortful language processes that depend on executive control and working memory, including verbal fluency and sentence-level comprehension. Verbal fluency tasks, in particular, are known to rely on both lexical retrieval and executive search processes (Shao et al., 2014), making them theoretically well-suited to detect exercise-related effects. Sentence comprehension was included given its reliance on working memory and domain-general cognitive control mechanisms (Ivanova et al., 2015; Schumacher et al., 2022). Together, this battery allowed us to examine whether exercise-related changes would manifest selectively in higher-level language functions supported by domain-general cognitive systems. Given the exploratory nature of this pilot study, the small sample size and the heterogeneity inherent to aphasia, we opted to analyze individual measures rather than compute composite scores, in order to preserve sensitivity to domain-specific patterns of change.

Detailed presentation of the physical fitness data is beyond the scope of the current paper; they are discussed in detail in our companion publication (Thompson et al., 2026). Here we limit our discussion of the physical fitness measures to exploring how changes in physical fitness are associated with changes in language and cognition. So, in the current study we focus on the following outcome measures.

### Language measures

- **Western Aphasia Battery – Revised (WAB)** (Kertesz, 2007) is a test of general language abilities in aphasia. Participants are assessed with 10 main language subtests, clustering into Spontaneous Speech, Auditory Verbal Comprehension, Repetition, and Naming/Word Finding subtest scores. Scores from these subtests comprise the WAB Aphasia Quotient (AQ), a general measure of aphasia severity. The WAB is one of the most commonly-used aphasia batteries and has been recommended as a core outcome measure for aphasia treatment studies by the recent Research Outcome Measurement in Aphasia consensus statement (Wallace et al., 2019). Here we evaluate both the overall score (AQ) and the subtest scores.
- **Philadelphia Naming Test (PNT)** (Roach et al., 1996) is a well-recognized measure of naming ability that now has a computerized adaptive version (Hula et al., 2015, 2020) that reduces test length while maximizing measurement precision by providing a standardized T-score.
- **Curtiss-Yamada Comprehensive Language Evaluation-Receptive (CYCLE-R)** (Curtiss, 1988) is a test of sentence-level comprehension using an auditory sentence-picture matching task. The CYCLE-R is particularly sensitive to syntactic processing deficits, as it uses a very limited vocabulary while assessing comprehension of sentences of varying difficulty ranging from simple to complex multi-clause relatives. In the current study, we used an overall accuracy score to index performance (for more details see Biondo et al., 2024).
- **Category and Letter Verbal Fluency Tasks** stand at the intersection of language and cognition, as performance on them is influenced both by word retrieval and by executive control abilities (Shao et al., 2014). Participants are asked to name as many items as possible in 1 minute for a given category (animals) and for a given letter (“s”) with the number of words correctly produced for each condition recorded.
- **Hopkins Verbal Learning Test-revised (HVLT)** (Brandt & Benedict, 2001) is used to assess verbal memory and learning ability in various clinical populations (Andrews et al., 2014; Skidmore et al., 2010). The test requires the participant to memorize a list of 12 words from 3 different semantic categories across several trials. The Total Recall score, calculated as the sum of correct responses across three consecutive learning trials, was used to index performance.

### Cognitive measures

- **Visual Search task** evaluates focused attention and speed of processing by requiring participants to identify a target symbol in a collection of 5, 10, 15, or 20 symbols. Accuracy and reaction times were recorded.
- **Go/No-Go task** assesses inhibitory control with participants asked to respond with a keypress when a “Go” is presented on the screen but refrain from responding when a “No Go” is presented. The dependent measures were the participant’s hits – the proportion of correct responses on the Go trials – and their reaction times on hit trials.
- **Flanker task** (Eriksen & Eriksen, 1974) measures domain-general cognitive control, implicating both selective attention and inhibition. The task requires participants to respond to a central stimulus that is flanked by either congruent, incongruent, or neutral stimuli. Nonverbal stimuli (arrows) are used in the task with both accuracy and reaction times recorded.
- **Forward and Backward Digit Span Spatial Span tasks** measure verbal and spatial working memory respectively by requiring participants to recall increasing sets of numbers/location of tokens in forward and reverse order. Task performance was indexed by maximal span size.
- **Stroop task** measures executive control and inhibition abilities with participants identifying the color of the printed word. The presented words are the names of different colors. The task includes congruent trials, where the written word matches its font color, and incongruent trials, where they do not match. The dependent measures are the participant’s accuracy by condition (congruent or incongruent) and their reaction times on correct trials by condition.

### Physical fitness measures

- **2-minute Step Test** (Rikli & Jones, 1999) is our core functional measure of physical fitness, that explicitly evaluates aerobic (cardiovascular) endurance. The outcome measure was the number of marching steps (knee raises to a point midway between the kneecap and top hip bone) completed in 2 minutes.

Our physical fitness tests and cognitive assessments were administered during in-person assessment sessions, with the cognitive assessments always given first to eliminate possible acute effects of exercise. All of our cognitive tasks were programmed in PsyToolkit (Stoet, 2010, 2017) for automated presentation and scoring. They were administered on a 14-inch MacBook Pro computer during in-person assessment sessions. For further details on our cognitive tasks see Anderson et al. (2024). Our language assessments were administered remotely via Zoom. Previous work has shown validity of videoconference administrations for standardized language assessment tools with proper modifications, which were carefully followed in our study (Dekhtyar et al., 2020).

### Statistical analysis

For the first aim, we analyzed safety, adherence and participant satisfactions qualitatively. For the second aim, pre- and post-intervention language and cognitive scores were compared using two-tailed Wilcoxon signed-rank tests. This nonparametric approach was selected because of the small sample size and the non-normal distribution expected for several outcome measures. To evaluate effect sizes of these comparisons we used rank - biserial correlations. This effect size measure reflects the proportion of favorable pre-post pairs – that is, how often post-intervention scores exceed pre-intervention scores relative to the reverse. For the third aim, we explored the associations between improvements in language and cognitive measures that showed positive changes at an individual level in aim two and changes on the main physical fitness measure using non-parametric two-tailed Spearman correlations.

All data analyses were performed using R Statistical Software (ver. 4.3.1; R Core Team, 2021) and figures were drawn in ggplot2 (ver. 3.4.3; Wickham, 2016). All statistical analyses were conducted using two-tailed significance testing. Given the exploratory nature of this pilot feasibility study, we report uncorrected *p*-values here and emphasize effect sizes and individual change patterns rather than definitive hypothesis testing.

## Results

### Safety, adherence and satisfaction

The new HI-FIIV intervention was found to be safe for individuals with post-stroke aphasia. There were no documented adverse events during the intervention. One participant (P07) withdrew from the study after the 11^th^ class because of an aggravation of a prior musculoskeletal injury (unrelated to stroke) due to an incident that occurred outside of the study resulting in a 14.3% dropout rate. All subsequent analyses are conducted on *N* = 6 participants unless otherwise specified.

Attendance and adherence rates were exceptionally high. Participants attended all sessions, except one participant (P06) who missed 3 classes during week 4 of the intervention due to a planned family vacation. Thus, overall attendance rate across all participants who completed the study was 97.9%. Furthermore, participants carefully followed the exercise protocol and adhered to all the intervention requirements (i.e., performing certain movements, alternating between high-intensity and slow recovery exercises, exercising in weighted vests) demonstrating high compliance.

Finally, all participants subjectively reported that they greatly enjoyed the program, with an average satisfaction rating of 9.6 out of 10 on an anonymous post-exercise survey. At the conclusion of the program, all were eager to continue with the same workout regimen and were motivated to start attending exercise classes in their community. As one participant stated: “*It was a really wonderful experience! The next thing for me is to get another positive in a group to join an exercise program that I see people and it will be two or three times a week. That’s what I’m looking for. The next step that I have to do is get a group and start doing it more because the study was really wonderful for me*.” Following the intervention individuals with aphasia subjectively reported better physical shape, more energy, improvements in language and memory.

### Changes in language and cognitive measures

**Figure 3.**
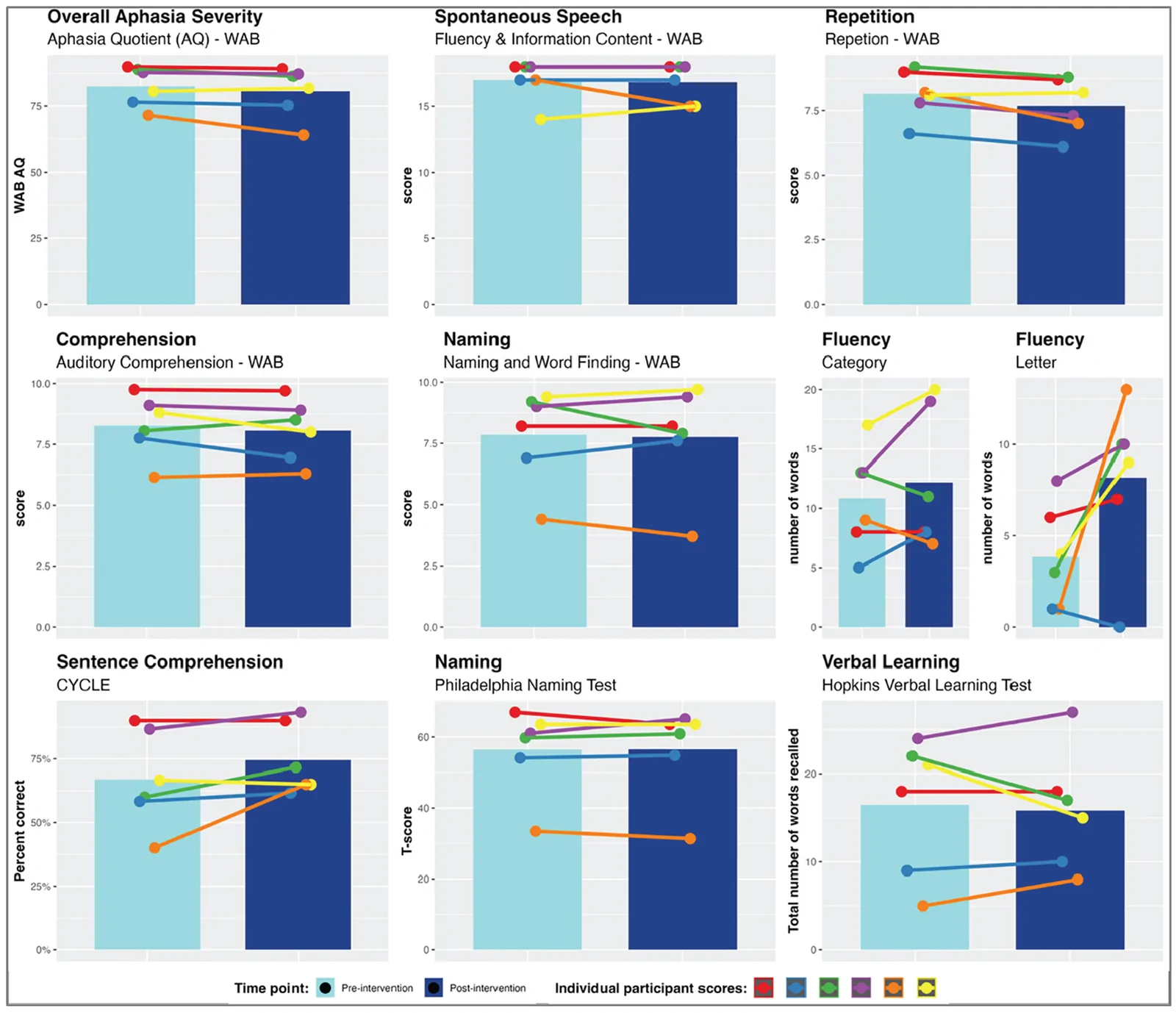
Participant language scores pre- and post-intervention. Bars represent group means at each time point, while individual points and connecting lines illustrate participant scores and their change trajectories. Each color represents an individual participant, with consistent color coding across panels. WAB – subtest score from the western aphasia battery.

**Figure 4.**
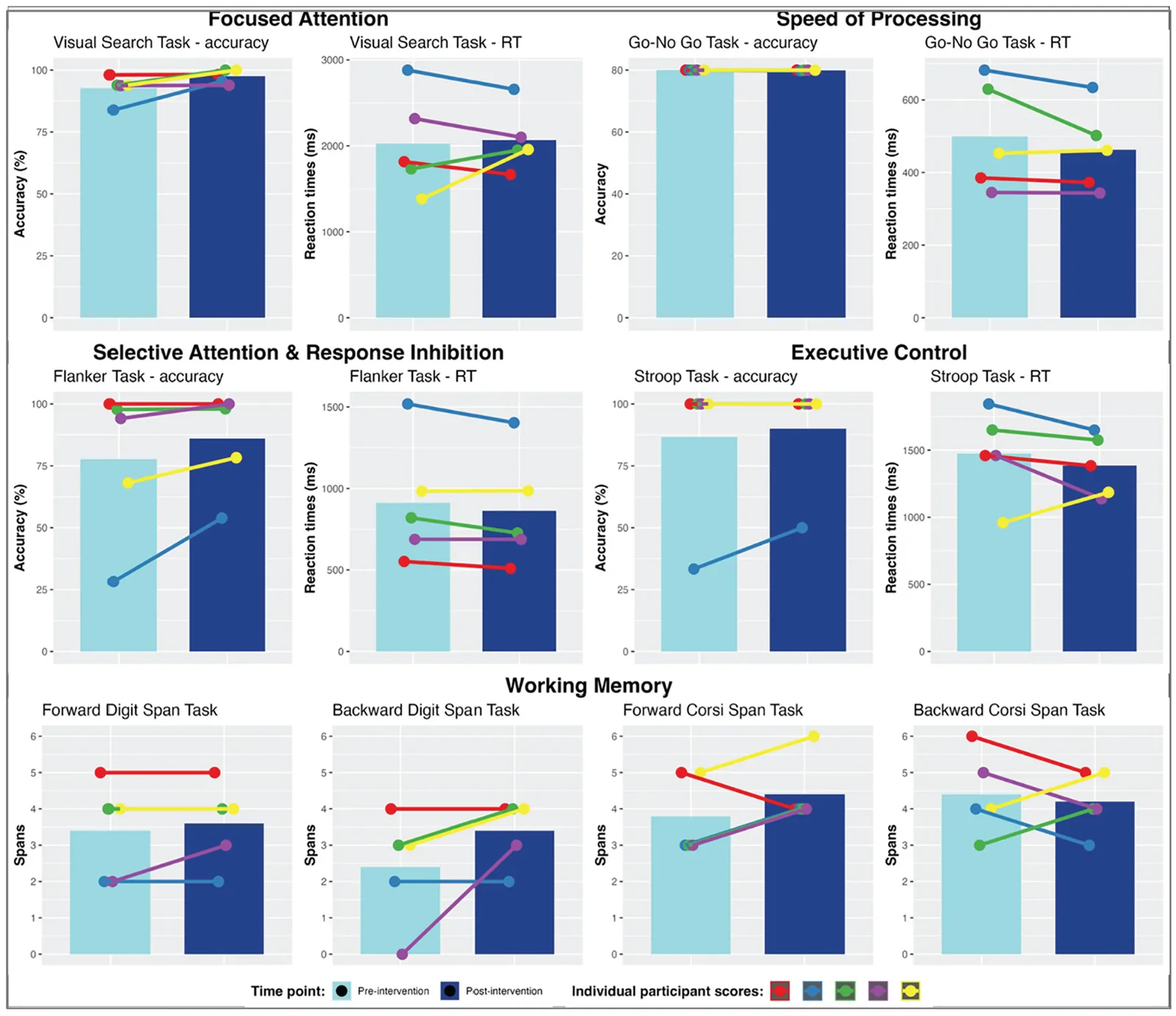
Participant performance on cognitive tasks pre- and post-intervention. Bars represent group means at each time point, while individual points and connecting lines illustrate participant scores and their change trajectories. Each color represents an individual participant, with consistent color coding across panels. Note that faster reaction times are reflected by lower values. RT – reaction time.

Descriptive statistics for language and cognitive measures before and after the intervention are presented in Tables 2 and 3, respectively. Group means at both time points, together with individual participant trajectories, are depicted in Figures 3 and 4. Note that participant P05 was unable to complete the cognitive tasks due to difficulty with understanding and remembering instructions, thus analyses of cognitive measures were performed with five participants.

Changes in language performance varied considerably across participants (Figure 3). The largest mean improvements were observed for letter fluency, with an average gain of 4.3 words (Table 2). Similar patterns of positive change were seen in sentence comprehension, as measured by the CYCLE-R, and category fluency. These effects were accompanied by moderate to large effect sizes, as indicated by rank-biserial correlations (r(rb)≥.6), and inspection of individual trajectories indicated improvements for the majority of participants, with variability in the magnitude of change across participants. Although these changes did not reach statistical significance under two-tailed testing, the pattern of results points to potential responsiveness of higher-level language functions to the intervention. By contrast, performance on WAB subtests, the PNT, and HVLT remained largely stable, suggesting that any observed changes were selective rather than global.

Similarly, cognitive measures showed substantial variability across participants (Figure 4). The most consistent positive changes were observed for Visual Search and Flanker task accuracy, reflecting possible gains in focused and selective attention (Table 3). Notably, for both measures the rank-biserial correlations were 1, indicating that all participants demonstrated positive changes following the intervention, although the magnitude of change varied. In addition, three of the four working memory tasks showed positive change, with the largest gains observed on the Digit Backward task. Overall, the strongest and most consistent gains were observed for tasks tapping focused and selective attention and working memory, with performance on other cognitive measures remaining largely stable. While these non-significant findings should be interpreted cautiously given the small sample size and exploratory analyses, the pattern of results suggests that attention and executive processes may be particularly promising targets for future study.

### Relationship between language and cognitive measures and changes in physical fitness

Lastly, we examined the relationship between changes in physical fitness and improvements in language and cognitive performance. We focused on measures that, as described above, showed patterns of change consistent with improvements, including Letter and Category Fluency, CYCLE-R, Visual Search accuracy, Flanker accuracy, and the Digit Backward and Corsi Forward span tasks.

As expected, participants demonstrated significant gains on the primary physical fitness measure of aerobic endurance, with average step count on the 2-minute step test increasing from 91 to 111 post-intervention (*W* = 21, *p* = .036, two-tailed). The effect size was large (*r(rb)* =1), indicating that all six participants improved their aerobic endurance. Broader findings on physical fitness from this cohort are reported in detail elsewhere (Thompson et al., 2026). In the current paper, we focus specifically on the relationship between this robust improvement in aerobic endurance and concurrent changes in language and cognition.

Spearman correlations revealed significant positive associations between gains in aerobic endurance and both letter fluency (*r*_*s*_ = .89, *p* = .019) and sentence comprehension (CYCLE-R, *r*_*s*_ = .83, *p* = .042). These findings suggest those who made more improvements in aerobic endurance also tended to make greater gains in fluency and sentence comprehension. Positive but nonsignificant correlations were also observed for working memory measures, including Digit Backward (*r*_*s*_ = .80, *p* = .111) and Corsi Forward spans (*r*_*s*_ = .71, *p* = .182). By contrast, no meaningful associations emerged for category fluency, Visual Search accuracy, or Flanker accuracy (*r*_*s*_ < .5, *p* > .2). These findings suggest that aerobic gains may preferentially support higher-level language and working memory functions.

## Discussion

The current study explored safety and feasibility of a new exercise intervention, HI-FIIV, for stroke survivors with aphasia and its potential to positively impact language and cognitive outcomes. The exercise program was designed on principles of high-intensity functional interval training and targeted multiple domains of physical fitness, including cardiovascular endurance, muscle strength, balance and mobility. Six out of seven participants with post-stroke aphasia successfully completed an eight-week-long, three-times-a-week in-person physical exercise intervention. We demonstrated that the HI-FIIV program is safe for stroke survivors with aphasia, showing high attendance rates and compliance with the intervention, along with high satisfaction rates. Following the intervention, beyond improvements in physical fitness, we also observed positive changes in specific cognitive and language domains, highlighting its potential in supporting aphasia recovery.

### Safety, adherence and satisfaction

The new HI-FIIV exercise program was found to be safe and feasible for individuals with post-stroke aphasia. In our 8-week study we observed no incidents concerning the safety of the HI-FIIV exercise program itself, with individuals with aphasia and variable motor impairments (including dense hemiparesis of upper and lower extremities) able to complete all the high-intensity exercises. The observed 14.3% dropout rate, resulting from one participant discontinuing participation due to a pre-existing musculoskeletal injury, was lower than the 18% average reported in other stroke exercise intervention studies (Dee et al., 2020; Marsden et al., 2013). Furthermore, PWA were consistently engaged and highly motivated to attend classes and follow through with the exercise regimen, demonstrating high attendance and adherence rates. In our companion publication focused on the feasibility and physiological effects of the intervention, we additionally explore intervention adherence in terms of target heart rates during different phases of the intervention and provide strong evidence that individuals with aphasia can exercise at high intensities (see Thompson et al., 2026). We also observed significant improvements in physical fitness further substantiating adherence and fidelity of the exercise intervention (see Thompson et al., 2026, for more details).

Lastly, individuals with aphasia enjoyed the exercise class immensely. All participants stated that they plan to sign-up for another exercise class and would join a similar exercise program in the future. There was unanimous agreement regarding the uniqueness of the HI-FIIV exercise program for stroke survivors and that they benefited from the high-intensity component of the intervention.

### Changes in language and cognition

Although preliminary, the results of this pilot study suggest that participation in the 8-week long HI-FIIV exercise program was associated with selective improvements in language and cognition in individuals with post-stroke aphasia. Specifically, participants demonstrated the most consistent positive changes in letter fluency, followed by category fluency and sentence-level comprehension. These findings are consistent with prior reports suggesting that exercise may enhance higher-order language processes, particularly those that place demands on both word retrieval and executive control (Ezeugwu & Manns, 2018; Harnish et al., 2018; Pyöriä et al., 2007); although, as stated in the beginning of this paper, findings on the impact of physical exercise on language deficits specifically in aphasia are very limited (Mayer et al., 2021). Interestingly, the observed changes were not global; standardized measures such as the WAB and the PNT remained stable, suggesting that exercise may preferentially support language tasks that rely on effortful retrieval and flexible cognitive control rather than basic naming or repetition. One possible explanation for this selective pattern is that high-intensity exercise might preferentially engage neural systems supporting executive control and cognitive flexibility, which are more strongly recruited during effortful language tasks such as verbal fluency and sentence comprehension than during more constrained tasks such as naming or repetition.

The cognitive outcomes further support this interpretation. A pattern of positive changes was observed in Visual Search and Flanker task accuracy, both of which index focused and selective attention. Additional consistent changes were noted in working memory measures, potentially pointing to a broader pattern of improvements in executive functions. These results are coherent with prior stroke studies reporting exercise-related improvements in attention, memory, and executive control (El-Tamawy et al., 2014; Kim & Yim, 2017; Kluding et al., 2011; Marzolini et al., 2012; Unibaso-Markaida et al., 2019). In turn, these domains, attention, working memory, and cognitive control, are widely recognized as critical for successful language production and comprehension (Bose et al., 2022; Choinski et al., 2020; Geranmayeh et al., 2014; Ivanova et al., 2015; Kiran & Thompson, 2019; Schumacher et al., 2022). This pattern raises the possibility that improvements in domain-general cognitive processes may underpin the selective language changes observed in this study, particularly for tasks that rely on both executive and linguistic processing. This hypothesis that cognitive improvements following exercise interventions may partially mediate the selective language gains should be tested in future, larger-scale studies.

Overall, across key measures (e.g., letter fluency, visual search, flanker accuracy), the majority of participants demonstrated improvement, although the magnitude of change varied. In all, our findings align with broader evidence that higher-intensity exercise promotes cognitive gains in stroke survivors (Gjellesvik et al., 2021; Liu-Ambrose et al., 2022; Mayer et al., 2021). The selective rather than generalized pattern of improvements mirrors prior studies demonstrating that exercise most robustly influences executive and attentional processes (Saunders et al., 2020; Vanderbeken & Kerckhofs, 2017). By extending this literature to include individuals with aphasia and including detailed language testing, the current study provides early evidence that high-intensity exercise may yield functional benefits that go beyond motor recovery and general cognition, enhancing the higher-level linguistic abilities.

Still clinical interpretation of the observed changes should be made cautiously. While a change of 5 points on the WAB Aphasia Quotient is often considered clinically meaningful (Hula et al., 2010; Rose et al., 2021), comparable minimal clinically significant difference values or minimal detectable change are not well established for the remaining language and cognitive measures, particularly in the aphasia population where psychometric benchmarks remain limited. As a result, the observed improvements should be interpreted as preliminary signals of potential responsiveness rather than definitive evidence of clinically meaningful change.

### Relationship between language and cognitive measures and changes in physical fitness

The relationship between physical fitness gains and improvements in language and cognition observed in this study provides further insight into potential mechanisms linking exercise to aphasia recovery. All participants demonstrated significant improvements in aerobic endurance, and those with the largest increases also showed the greatest gains in letter fluency and sentence comprehension. These associations are consistent with previous reports demonstrating that improvements in aerobic capacity are positively correlated with cognitive gains in stroke survivors (Kluding et al., 2011; Marzolini et al., 2012). Extending this work, our findings suggest that aerobic fitness may also support higher-level language outcomes that depend on executive resources, such as verbal fluency and complex sentence processing.

As discussed in the Introduction, several neurobiological mechanisms may link aerobic exercise to recovery processes in aphasia. The observed associations in the present study are broadly consistent with these proposed pathways. Aerobic exercise has been shown to increase cerebral blood flow and angiogenesis, supporting neural recovery in perilesional areas (Lucas et al., 2015; Smith & Ainslie, 2017). Increase in BDNF and IGF-1 following exercise can also enhance synaptic plasticity, neurogenesis, and cognitive resilience (Erickson et al., 2011; Mang et al., 2013; Ploughman & Kelly, 2016). Importantly, post-stroke studies have demonstrated that upregulation in circulating BDNF and IGF-1 levels following high-intensity aerobic exercise (Ashcroft et al., 2022; Hsu et al., 2021) correlate with cognitive and motor improvements (El-Tamawy et al., 2014; Saucedo Marquez et al., 2015). Improvements in higher-level linguistic abilities, attentional control and working memory observed here may represent intermediate outcomes of these physiological processes. The absence of strong associations between fitness gains and some measures (e.g., category fluency, Visual Search and Flanker accuracy) highlights that not all domains are equally sensitive to aerobic improvements, and further research is needed to delineate which aspects of cognition and language benefit most from exercise-induced physiological changes. Although, due to small sample size, all these effects (and lack thereof) should be interpreted with caution.

Still taken together, these preliminary results support the hypothesis that physical fitness gains are not merely coincidental with language improvements but may play a contributory role in amplifying the recovery of linguistic and cognitive functions. Future studies with larger samples and mechanistic measures, such as neuroimaging of cerebral blood flow, structural/functional connectivity, and biomarkers of neurotrophic activity, are warranted to clarify causal pathways and to identify the subgroups of people with aphasia most likely to benefit from exercise-based interventions.

### Limitations

This study has several important limitations. First, as a small pilot feasibility trial with only seven participants, our findings should be interpreted cautiously and cannot be generalized to the broader population of individuals with post-stroke aphasia. The limited sample size also restricted our ability to fully explore heterogeneity in response to the intervention. Second, participants in this study primarily presented with mild-to-moderate aphasia. Thus, the feasibility, safety, and adherence of the HI-FIIV program for individuals with more severe language impairments remains unknown. Similarly, the intervention in its current format is designed for participants who are independently ambulatory, limiting applicability to those with more profound motor impairments. Future adaptations of the program will need to determine how to safely accommodate individuals with greater mobility restrictions.

Third, our outcome measures did not include assessments of psychological or psychosocial well-being (e.g., depression, anxiety, or quality of life), despite strong evidence that regular exercise can positively impact these domains (Zhang et al., 2025). Incorporating such measures will be important for future work to provide a more comprehensive picture of how high-intensity exercise may benefit people with aphasia. Fourth, lack of neuroimaging or biomarker measures limit insights into possible neurophysiological mechanisms of observed language and cognitive treatment gains.

Finally, the single-group pre/post design precludes strong causal inferences. Without multiple baseline assessments, a crossover design, or a parallel control group, it is difficult to disentangle intervention-related gains from possible spontaneous recovery, practice effects, or variability over time. However, this design was intentionally selected for this Phase 1 pilot feasibility study, consistent with early-phase behavioral intervention development frameworks, where the primary goals are to establish safety, adherence, and intervention fidelity, and to identify outcome measures and effect size estimates to inform subsequent trials. Accordingly, our findings regarding language and cognitive benefits should be considered preliminary and hypothesis-generating rather than confirmatory. Still, taken together with promising findings on the safety, adherence, and feasibility of the novel intervention, these limitations underscore the need for larger randomized controlled trials with more diverse samples, comprehensive psychosocial and neurophysiological outcome measures, and rigorous experimental designs to establish the efficacy of the HI-FIIV program for supporting aphasia recovery. Future work, including our ongoing randomized controlled trial, will directly address these limitations by incorporating appropriate control conditions to evaluate efficacy and underlying mechanisms.

## Conclusions

This Phase 1, proof of concept, pilot feasibility study demonstrated that individuals with aphasia can safely and successfully participate in a high-intensity functional interval training program. The HI-FIIV design effectively accommodated individuals with a range of moderate to mild motor and language impairments, resulting in excellent adherence, high satisfaction, and meaningful improvements in physical fitness. Importantly, participants exhibited potentially promising gains in sentence-level comprehension and verbal fluency, along with enhancements in attention and working memory, suggesting that exercise may support language recovery through improvements in domain-general cognitive processes. Our exploratory analyses further suggest that aerobic fitness gains may play a contributory role in these outcomes. Exercise-induced improvements in cardiovascular endurance are thought to promote cerebral blood flow, angiogenesis, and upregulation of neurotrophic factors such as BDNF and IGF-1, which together support neuroplasticity and recovery after stroke. These mechanisms may underlie the observed associations between aerobic endurance and gains in higher-level language and cognitive functions.

Taken together, these findings afford proof-of-concept that high-intensity exercise interventions are both feasible and potentially beneficial for people with post-stroke aphasia. Specifically, results of the current study provide robust support for the safety, feasibility and fidelity of the novel HI-FIIV intervention. By targeting physiological, motor, cognitive, and linguistic systems simultaneously, the HI-FIIV program addresses a critical clinical gap and offers an accessible adjuvant intervention for people living with post-stroke aphasia. Given the current promising results, the HI-FIIV program is well-positioned for successful implementation in larger clinical trials aimed at further elucidating its comprehensive impact on aphasia recovery.

## Figures and Tables

**Table 1.** Individual participant characteristics.

ID	Age (years)	Gender	Post-onset* (months)	Education	Etiology	Motor impairments	WAB AQ^#^	Aphasia Type•
P01	58	F	87	MBA	LH hemorrhagic stroke, Moyamoya disease	Right-sided hemiparesis, basal ganglia involvement	89.9	Anomic
P02	62	F	65	MEdu	LH ischemic stroke, RH ischemic stroke 4 years later	None	76.5	Conduction
P03	62	M	241	MS	LH stroke due to carotid artery dissection	Dense right-sided hemiparesis	88.9	Anomic
P04	46	F	11	BA	RH stroke due to carotid artery dissection	None	87.8	Anomic
P05	62	M	26	MS	LH stroke with diffuse bilateral hypoxic injury due to cardiac arrest	None	71.5	Transcortical sensory
P06	47	F	54	MS	LH ischemic stroke	Minor right-sided weakness	80.6	Anomic
P07	57	F	46	10-th grade	LH ischemic stroke, RH ischemic stroke 2 years later followed by a small LH ischemic stroke 3 months later	None	79.1	Conduction

* Time post-onset from the first stroke.

# Western Aphasia Battery Aphasia Quotient at pre-intervention.

• Aphasia type based on the WAB classification at pre-intervention.

**Table 2.** Descriptive statistics, Wilcoxon signed-rank tests, and effect sizes for language measures pre- and post-intervention (N = 6).

Measure	Measure-ment units	Pre-intervention *M (SD)*	Post-intervention *M (SD)*	Mean Differe-nce (Post – Pre)	Pre-intervention score range	Post-intervention score range	Wilcoxon *W*	*p*-value	Rank-biserial *r*
Spontaneous Speech (WAB)	score	17 (1.55)	16.83 (1.47)	−0.17	14–18	15–18	1.0	1.000	−0.33
Repetition (WAB)	score	8.15 (0.93)	7.68 (1.06)	−0.47	6.6–9.2	6.1–8.8	1.0	0.059	−0.91
Naming & Word Finding (WAB)	score	7.85 (1.92)	7.75 (2.15)	−0.10	4.4–9.4	3.7–9.7	6.0	0.787	−0.20
Auditory Verbal Comprehen-sion (WAB)	score	8.27 (1.26)	8.06 (1.26)	−0.21	6.2–9.8	6.3–9.7	6.0	0.402	−0.43
WAB AQ	score	82.53 (7.54)	80.65 (9.55)	−1.88	71.5–89.9	64–89.2	3.5	0.172	−0.67
PNT – total score	T-score	56.5 (12.07)	56.56 (12.87)	0.07	33.5–67	31.4–65.1	8.0	1.000	0.07
Fluency – category	number of words	10.83 (4.31)	12.17 (5.85)	1.33	5–17	7–20	12.0	0.276	0.60
Fluency – letter	number of words	3.83 (2.79)	8.17 (4.45)	4.33	1–8	0–13	19.5	0.074	0.86
CYCLE-R – total score	proportion correct	0.67 (0.19)	0.74 (0.14)	0.07	0.4–0.9	0.6–0.9	14.0	0.106	0.87
HVLT – total recall	number of words	16.5 (7.71)	15.83 (6.74)	−0.67	5–24	8–27	6.0	0.786	−0.20

WAB – Western Aphasia Battery. PNT – Philadelphia Naming Test. CYCLE-R – Curtiss-Yamada Comprehensive Language Evaluation-Receptive. HVLT – Hopkins Verbal Learning Test.

**Table 3.** Descriptive statistics, Wilcoxon signed-rank tests, and effect sizes for cognitive measures pre- and post-intervention (N = 5).

Measure	Measure-ment units	Pre-intervention M (SD)	Post-intervention M (SD)	Mean Differe-nce (Post – Pre)	Pre-intervention score range	Post-intervention score range	Wilcoxon W	*p*-value	Rank-biserial *r*
Visual Search – accuracy (%)	percent correct	92.7 (5.26)	97.52 (2.74)	4.82	83.9–98.1	93.9–100	15	0.059	1.00
Visual Search – RT	ms	2025.78 (583.08)	2066.58 (365.9)	40.80	1383.2–2881.4	1667.3–2657.8	8	1.000	0.07
Go/No-Go – hits	number of hits	80 (0)	79.96 (0.09)	−0.04	80–80	79.8–80	0	1.000	−1.00
Go/No-Go – RT	ms	499.06 (149.53)	462.77 (115.44)	−36.29	345–682.1	343.4–634.3	2	0.178	−0.73
Flanker – accuracy (%)	percent correct	77.65 (30.45)	86.05 (20.17)	8.39	28.2–100	53.9–100	10	0.100	1.00
Flanker – RT	ms	912.59 (374.43)	862.9 (346.56)	−49.70	552.4–1518.3	510.2–1403.1	3	0.281	−0.60
Stroop congruent – accuracy (%)	percent correct	86.67 (29.81)	90 (22.36)	3.33	33.3–100	50–100	1	1.000	1.00
Stroop congruent – RT	ms	1324.59 (323.39)	1288.9 (257.12)	−35.69	866.3–1731.4	953.7–1542.4	7	1.000	−0.07
Stroop incongruent – accuracy (%)	percent correct	87.11 (17.87)	84.87 (25.25)	−2.24	57.8–100	40–100	6	0.787	−0.20
Stroop incongruent – RT	ms	1624.85 (345.74)	1483.59 (206.95)	−141.26	1053.6–1956.7	1266.7–1756.4	4	0.418	−0.47
Digit Forward – span	maximal span	3.4 (1.34)	3.6 (1.14)	0.20	2–5	2–5	1	1.000	1.00
Digit Backward – span	maximal span	2.4 (1.52)	3.4 (0.89)	1.00	0–4	2–4	6	0.174	1.00
Corsi Forward – span	maximal span	3.8 (1.1)	4.4 (0.89)	0.60	3–5	4–6	12	0.233	0.60
Corsi Backward – span	maximal span	4.4 (1.14)	4.2 (0.84)	−0.20	3–6	3–5	6	0.766	−0.20

RT – reaction time.

## Data Availability

All data produced in the present study are available upon reasonable request to the authors.

## Appendix

**Table A1.** Detailed exercise components and durations in the HI-FIIV program.

				Duration (seconds)		
Phase		Type of Exercise	Exercises	Week 1	Week 2	Week 3
**Warm-up**		Seated (6 minutes) – dynamic joint mobility	Deep Breathing Meditation		120	
			Seated Arm Hugs		60	
			Seated Hip Steps		60	
			Seated Ankle Rotations		60	
			Seated Ankle Point/Flex		60	
		Standing (4 minutes)	March (with chair if needed)		120	
			Hip Hinge with Arm Swing		60	
			Torso Rotation Punches		60	
**HIIT Stage 1 – Weeks 1–3** (Repeated 3 Times)	HIIT Block 1	**HI #1**	**Unweighted Hip Hinge**	30	45	60
		Rec #1	Rocking Feet at Chair - Sagittal		60	
	HIIT Block 2	**HI #2**	**Marching with Scapular Squeeze**	30	45	60
		Rec #2	Rocking Feet at Chair - Frontal		60	
	HIIT Block 3	**HI #3**	**Forward/Backward Stomps at Chair**	30	45	60
		Rec #3	Narrow Stance Forward Reach		60	
	HIIT Block 4	**HI #4**	**Sit to Stands**	30	45	60
		Rec #4	Narrow Stance Rotations		60	
**HIIT Stage 2** (Repeated 3 times) **Weeks 4–6**	HIIT Block 1	**HI #1**	**Weighted Hip Hinge**	45	60	60
		Rec #1	Step/Return at Chair - Sagittal		60	
	HIIT Block 2	**HI #2**	**Marching with Horizontal Abduction**	45	60	60
		Rec #2	Step/Return at Chair - Frontal		60	
	HIIT Block 3	**HI #3**	**Forward/Backward Stomps at Chair**	45	60	60
		Rec #3	Narrow Stance Multidirect Reach		60	
	HIIT Block 4	**HI #4**	**Sit to Stands Arms on Chest**	45	60	60
		Rec #4	Narrow Stance X-Cross Rotations		60	
**HIIT Stage 3** (Repeated 3 Times) **Weeks 7–8**	HIIT Block 1	**HI #1**	**Weighted Hip Hinge Arm Swing**	60	60	–
		Rec #1	Step/Return - Sagittal		60	
	HIIT Block 2	**HI #2**	**Marching Scaption**	60	60	–
		Rec #2	Step/Return - Frontal		60	
	HIIT Block 3	**HI #3**	**Lateral Stomps at Chair**	60	60	–
		Rec #3	Split Stance Multidirect Reach		60	
	HIIT Block 4	**HI #4**	**Sit to Stands**	60	60	
		Rec #4	Split Stance X-Cross Rotations		60	
**Cooldown**		Standing (2 minutes)	Hip Rotations		60	
			Chair Hip Hinge with Deep Breathing		60	
		Seated (4 minutes) dynamic joint mobility	Seated Arm Hugs		60	
			Seated Hip Steps		60	
			Seated Ankle Rotations		60	
			Deep Breathing Meditation		60	

HI – high-intensity exercises, Rec – recovery exercises.
